# When in doubt, Google it: distress-related information seeking in Italy during the COVID-19 pandemic

**DOI:** 10.1186/s12889-021-11887-2

**Published:** 2021-10-20

**Authors:** Dario Monzani, Laura Vergani, Giulia Marton, Silvia F. M. Pizzoli, Gabriella Pravettoni

**Affiliations:** 1 Department of Oncology and Hemato-Oncology, University of Milan, Via Festa del Perdono 7, 20122 Milan, Italy; 2 Applied Research Division for Cognitive and Psychological Science, IEO, European Institute of Oncology IRCCS, Milan, Italy

**Keywords:** COVID-19, Infodemiology, Psychological distress, Anxiety, Depression, Insomnia, Google

## Abstract

**Background:**

Psychological health has been one of the aspects affected by the recent COVID-19 pandemic. We aim to evaluate the patterns of Google search for mental distress symptoms of Italian citizens during the various phases of the COVID-19 pandemic.

**Methods:**

We assessed Google searches for psychological-health related words. We gathered and analyzed data on daily search queries on depression, anxiety, and insomnia from Google Trends, in a time ranging from the Pre-COVID phase (beginning 25th January 2020) up to the second wave phase (ending 17th October 2020). We performed three general linear models on search trends of the three words and tested whether and to what extent official data about new cases of COVID-19, information searching on new cases, and the government health measures impacted on these trends.

**Results:**

Average daily search queries were higher for anxiety, followed by depression and insomnia. General linear models performed to assess differences in daily search queries for anxiety, depression and insomnia were significant, respectively [F(13, 253) = 6.80, *P* < .001]; [F(13, 253) = 10.25, *P* < .001]; [F(13, 253) = 6.61, *P* < .001].

Specifically, daily search queries differed among different phases of managing the COVID-19 outbreak: anxiety [F(5, 253) = 10.35, *P* < .001, ${n}_p^2$ = .17]; depression [F(5, 253) = 13.59, *P* < .001, ${n}_p^2$ = .21]; insomnia [F(5, 253) = 3.52, *P* = .004, ${n}_p^2$ = .07].

**Conclusions:**

Our study contributed to the investigation of online information-seeking behaviors of Italians regarding mental health throughout the entire phase of the pandemic and provides insights on the possible future trends of mental distress during upcoming pandemic phases.

**Supplementary Information:**

The online version contains supplementary material available at 10.1186/s12889-021-11887-2.

## Introduction

The ongoing coronavirus disease 2019 (COVID-19) pandemic is heavily affecting all aspects of our lives. Italy was the first Western country heavily affected by the pandemic. As of the end of October 2020, 679,430 people had been tested positive for Covid-19, while deaths since the beginning of the Pandemic totaled 38,618 [1]. At the time of writing (March 2021), Italy is the seventh country most affected worldwide per total number of cases [2]. COVID-19 is causing “psychological and social effects” that can “affect mental health now and in the future” [3]. In this unprecedented situation, psychological issues, public health and policies have been identified as areas of research that are fundamental to investigate [4]. It is indeed essential to rapidly understand the effects that the pandemic is having on mental health. More efficient methodologies are required to acquire a comprehensive knowledge of the situation in each country and each phase of the pandemic.

Nowadays, one in two European citizens searches online for health-related information [5], while the Internet is considered a source of valuable knowledge and advice also for mental health issues [6]. So, the saying “when in doubt, google it”, seems readily applicable to seeking out mental health information, regarding, for example, depression, anxiety, and insomnia. Every time we “google it”, we leave a digital footprint, constituting a sum of numerous pieces of valuable information that can easily and rapidly be used for research [7]. This approach based on online data has been defined as infodemiology, namely “the science of distribution and determinants of information in an electronic medium, specifically the Internet, or in a population, with the ultimate aim to inform public health and public policy” [8]. As Eysenbach [8] explained, one of its applications is the use of search engine queries on a topic to collect insights into people’s behaviors and their information needs.

Today, Google is the most used search engine worldwide [9] and in Italy, where it obtains a market share of 95.73% (February 2021) [10]. Its usage has been increasing sharply during this pandemic period [11]. Using Google data has proven to be a reliable method to study health-related behaviors, focusing on different topics [12]. Search queries on Google have been used to study diurnal variations in information seeking about depression: this help-seeking has specific patterns during the day, with a peak during the evening and night [7]. Moreover, search query volumes for depression-related symptoms followed weekly pattern with highest information seeking on Sundays [13]. Other studies were conducted in the COVID-19 pandemic context [14–16] and showing the feasibility of using Google Trends to monitor infectious diseases and COVID-19 [17].

Mental health in connection with COVID-19 has been targeted as well [18, 19]. Google searches for worry and anxiety-related terms increased in the US after the World Health Organization declared the pandemic in March 2020 [20]. In the Italian context, Google search for hygiene-related terms and emotional terms have been considered [21]. These results highlighted an overall increase in Web interest in COVID-19 only when this became direct national issue [21]. Specifically, Web search for generic news related to the virus was positively influenced by the number of COVID-19 cases at the national but not regional or international levels [21]. Moreover, while interest related to hygiene and prevention rose sharply during the first 3 months of the COVID-19 outbreak in Italy, search for emotional terms, such as “fear” and “stress”, displayed a slower growth [21]. Others found that the peak of search queries for “psychological support” in recent years was reached in March and April during the national lockdown [22]. However, no study has ever been conducted in Italy using Google Trends to monitor psychological distress (PD), in terms of anxiety, depression, and insomnia, during the pandemic.

## Objective

Our study aimed at monitoring PD in the Italian population during the ongoing COVID-19 pandemic by considering search engine data, namely Google Trends. We focused on depression, anxiety, and insomnia, as mental health dimensions proved to be affected by the pandemic [23]. We aimed at understanding whether PD differed among periods characterized by different restrictions taking place to limit the diffusion of the COVID-19. Italy was the first Western country to be hit by the COVID-19 pandemic, and specific public health measures and restrictions were implemented by the national government to limit the spread of the virus. Specifically, we considered six phases of managing the COVID-19 outbreak, each with specific public health measures, restrictions, and limitation of activities (for a brief overview, see [24, 25]):

- Pre-COVID (25th January 2020 -20th February 2020). On 25th January 2020, the first health protection action was taken by the government (public health checkpoints in Italian airports, for passengers arriving from China) [25]. On the 31st January 2020, after the first two confirmed cases in Italy (a couple coming from Wuhan), the Italian government declared a state of Emergency [26];
- COVID-19 outbreak (21st February 2020 -8th March 2020). On 21st February 2020, the Italian National Health Institute (*Istituto Superiore di Sanità)* confirmed the first indigenous case of COVID-19 (i.e., Italian “patient one” being tested positive in the northern Italian province of Lodi) [27]. Further clusters of infections in Northern Italy were subsequently found, and emergency public health measures were implemented, such as strict lockdown for eleven municipalities in Lombardy and Veneto and other restrictions at the national level (e.g., suspension of public events and school closure) [25]. On 8th March, a lockdown was proclaimed for Lombardy and other northern areas [28].
- Phase 1 (9th March 2020 -3rd May 2020). On 9th March 2020, Prime Minister Giuseppe Conte with the #iorestoacasa (#IStayAtHome) decree [29], proclaimed a national lockdown in Italy with strict restrictions to social gatherings and mobility, such as the prohibition against leaving homes unless for work needs or emergencies, limitation of travel and leisure activities, social distancing, and suspension of all “non-indispensable” commercial activities.
- Phase 2 (4th May 2020 -14th June 2020), as the epidemic curve slowed down, the Italian government issued another decree on the 4th May [30], starting a gradual loosening of restrictions: factories, restaurants, and gyms re-opened, but it was still mandatory to practice social distancing and wear masks.
- Phase 3 (15th June 2020 -15th August 2020), characterized by a further loosening of public health measures and restrictions (e.g., the re-opening of theaters and cinemas), as established by the decree issued on 11th June [31].
- Second wave (16th August 2020 -17th October 2020), as the epidemic curve started to slowly increase again, new restrictions (e.g., forbidding gatherings and incentivizing remote working) were set in place. On 16th August 2020, a decree issued by the Minister of Health required the closure of discos and nightclubs [32].

Moreover, we also evaluated the effect of daily official data about the number of new cases of COVID-19 and online information-seeking behavior related to this number. Specifically, we explored whether people’s daily information-seeking behaviors about PD were related to the actual spreading of the virus and the daily prevalence of people searching for information about it.

## Methods

Daily search data in Google was retrieved through Google Trends, a public website, free of charge, which analyzes the popularity of search queries in Google Search across time, countries, and languages. As Google is by far the most used search engine in Italy [10], we decided to rely solely on this. We collected data on daily search queries as relative search volumes (RSVs). RSV is adjusted by changes in Internet access over time: it is the ratio of a search query to the sum number of searches of all queries on a specific day [7, 33, 34]. This ratio is then rescaled on a 0–100 range, where 100 is the day with the highest search proportion for the considered time. Thus, RSV gives information on a specific Google query as its relative popularity to all queries in a given time frame. Since Google Trends provides daily data for the query period shorter than 9 months, we collected data about Italian search queries from 25th January (i.e., almost 1 month before Italian “Patient One” was tested positive) to 17th October 2020 to gain a clear picture of variations in PD from before the COVID-19 outbreak Italy to the subsequent phases of the pandemic. We extracted RSVs about search queries related to PD and daily information-seeking about new daily cases COVID-19.

Specific search terms considered were:

- ansia (the Italian translation for “anxiety”);
- depressione (the Italian translation for “depression”);
- insonnia (the Italian translation for “insomnia”);
- casi covid (the Italian translation for “covid cases”)
- casi coronavirus (the Italian translation for “coronavirus cases”).

Specifically, we choose to rely on two search terms (i.e., “casi covid”, “casi coronavirus”) for new cases because in Italy the term “covid” and “coronavirus” are used interchangeably. The daily mean of “casi covid” and “casi coronavirus” RSVs was used as an index of daily information seeking regarding new cases of COVID-19.

Search terms considered were all in the Italian language, not in other languages or regional dialects. Moreover, we considered only RSVs related to Italy as a whole, not taking into account regional variations.

Regarding PD, we chose to focus only on broad search terms (e.g., anxiety) and not more specific terms (e.g., anxiety symptoms, or anxiety symptomatology). This was because, after some preliminary searches, we found that broad search terms were searched more often than more specific ones. For example, on 21st June 2020, while the RSV for “depressione” (the Italian translation for depression) was 100, the RSV for “depressione sintomi” (the Italian translation for “depression symptoms”) was 3. This trend was the same for the whole period considered and for all the search terms (see, for example, https://trends.google.it/trends/explore?date=2020-01-25%202020-10-17&geo=IT&q=depressione).

Finally, we also retrieved official data about daily new cases of COVID-19 (i.e., “nuovi_positivi”) from the Italian Prime Minister's Office GitHub webpage [35]. For the frequency of search terms considered, as reported on the Google Trends website, please consult Additional file 1.

Data were used in compliance with the ethical guidelines for Internet Research [36]. The EU General Data Protection Regulation (EU-GDPR) 2016/679 provides for the use of anonymous data for research purposes under certain conditions. Since all analyses were performed on public and anonymized meta-data, no Institutional Review Board (IRB) approval was required for the completion of this study.

### Statistical analysis

Statistical analyses were conducted from 29th October to 5th November 2020. We performed three general linear models with Jamovi 1.1 [37, 38], one for each of three RVSs about search query of anxiety, depression, and insomnia. In each model, daily RVS was entered as the dependent variable. Daily official data about new cases of COVID-19 and information-seeking about new cases of COVID-19 were entered as continuous independent variables, while the phase of managing the COVID-19 outbreak was entered as a categorical independent variable (coded as, 1 = Pre-COVID; 2 = COVID-19 outbreak; 3 = Phase 1; 4 = Phase 2; 5 = Phase 3; 6 = Second wave). Since available evidence showed weekly pattern of online information seeking about PD [13], the day of the week was entered in each model to control for any weekly variations in anxiety, depression, or insomnia. Specifically, this effect assessed whether people were more likely to search for PD people on specific days of the week. All continuous independent variables were mean-centered. The magnitude of each effect was interpreted through its associated partial eta squared (i.e., ${\eta}_p^2;$ weak: .01 < ${\eta}_p^2$ ≤ .06; moderate:.06 < ${\eta}_p^2$ ≤ .14; strong: ${\eta}_p^2>.14\Big)$.

## Results

**Fig. 1:**
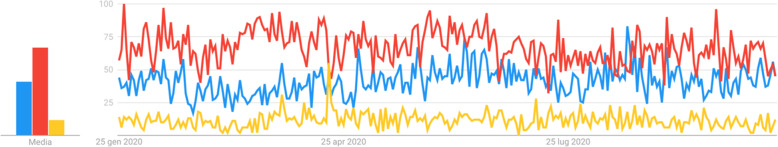
Means and frequency of RSVs for anxiety (in red), depression (in blue), and insomnia (in yellow) among the period considered. The figure was extracted directly from the Google trends website

RSVs mean and frequency for PD-related search terms - as extracted directly from the Google Trends website - during the period considered are reported in Fig. 1.

Average RSV for daily search queries for anxiety was the highest (*M* = 67), followed by daily search queries for depression (*M* = 41) and insomnia (M = 12). RSVs frequency for the anxiety search remained the highest across the whole period, with very few exceptions, in which depression was reported as being the most searched term. Insomnia remained the term searched less often for almost all the days considered.

**Fig. 2:**
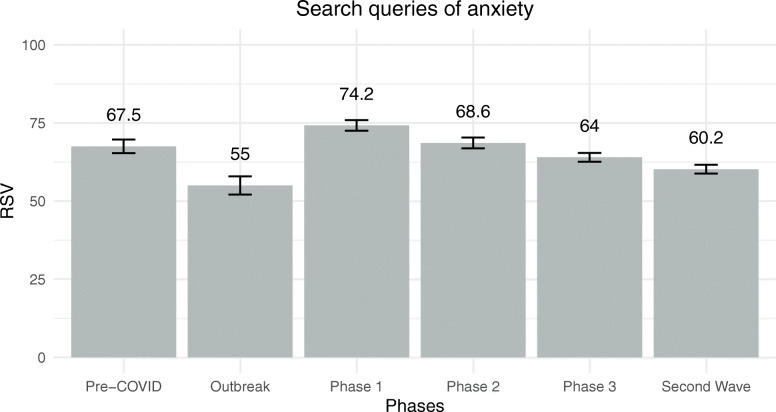
Estimated marginal means of RSVs for anxiety among the six phases of COVID-19 outbreak management

The general linear model performed to assess differences in daily search queries of anxiety was significant [*F*(13, 253) = 6.80, *p* < .001] and explained 26% of the dependent variable. RSVs were not related to daily new cases of COVID-19 [*F*(1, 253) = 2.04, *p* = .16, ${\upeta}_{\mathrm{p}}^2$ = .01, *β* = − .13] neither to daily search queries for new cases of COVID-19 [*F*(1, 253) = 0.05, *p* = .82, ${\eta}_p^2$ = .00, *β* = − .02]. Daily search queries of anxiety differed strongly among different phases of managing the COVID-19 outbreak [*F*(5, 253) = 10.35, *p* < .001, ${\eta}_p^2$ = .17]. Estimated marginal means of daily search queries for anxiety for each phase are reported in Fig. 2. Specifically, as highlighted by post hoc analyses with a Bonferroni correction, the daily RSV for anxiety was higher before COVID-19 than during the COVID-19 outbreak. Then, during the outbreak of COVID-19, people were less likely to search for anxiety than during Phase 1 and Phase 2. Moreover, RSV during Phase 1 was higher than RSV of Phase 3 and the second wave. Similarly, people googled anxiety more during Phase 2 than during the second wave. Finally, the days of the week were not associated with differences in daily search queries of anxiety [*F*(6, 253) = 1.74, *p* = .11, ${\eta}_p^2$ = .04].

**Fig. 3:**
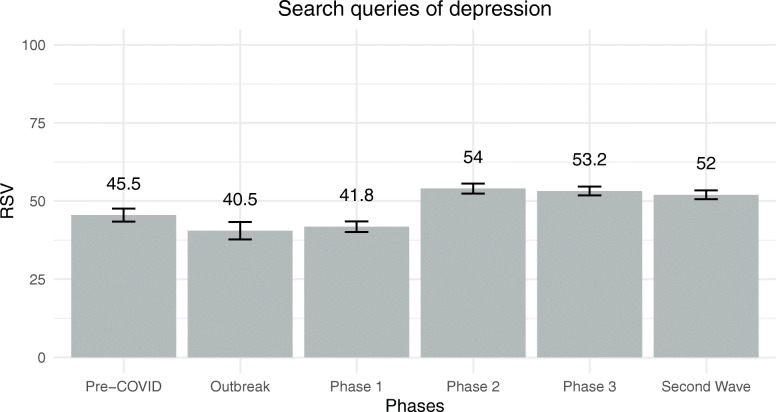
Estimated marginal means of RSVs for depression among the six phases of COVID-19 outbreak management

The model assessing differences in daily search queries for depression was significant [*F*(13, 253) = 10.25, *p* < .001] and explained 35% of the dependent variable. RSVs were not related to daily official new cases [*F*(1, 253) = 0.716, *p* = .398, ${\eta}_p^2$ = .00, *β* = − .07], while they were negatively but weakly related to daily search queries for new cases of COVID-19 [*F*(1, 253) = 4.27, *p* = .040, ${\eta}_p^2$ = .02, *β* = − .17]. Daily search queries for depression differed strongly among phases of managing the COVID-19 outbreak [*F*(5, 253) = 13.59, *p* < .001, ${\eta}_p^2$ = .21]. Daily RSVs for depression for each of the six phases of COVID-19 outbreak management are reported in Fig. 3. Daily RSVs for depression were lower before COVID-19 than during Phase 2 and Phase 3. Then, during the outbreak of COVID-19, people were less likely to search for depression on Google than during Phase 2 and Phase 3. Moreover, RSVs during Phase 1 were lower than RSVs of Phase 2, Phase 3, and second wave. Finally, the days of the week were moderately associated with differences in daily search queries of depression [*F*(6, 253) = 3.10, *p* = .006, ${\eta}_p^2$ = .07]. Specifically, as highlighted by post hoc analyses with a Bonferroni correction, people are more likely to use Google to search for depression on a Sunday than on a Wednesday.

**Fig. 4:**
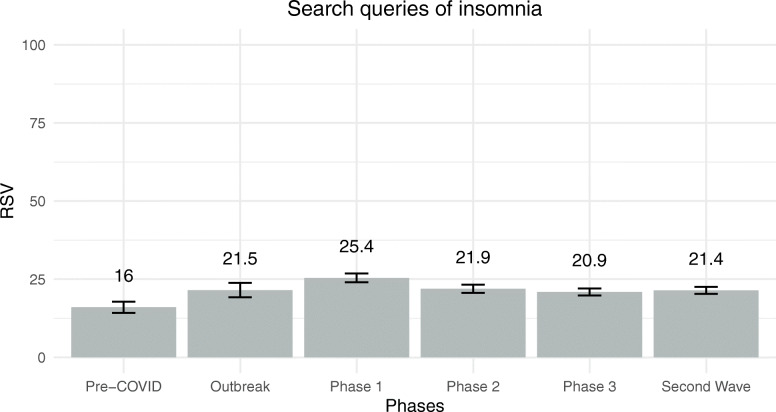
Estimated marginal means of RSVs for insomnia among the six phases of COVID-19 outbreak management

The model evaluating differences in daily search of insomnia was significant [*F*(13, 253) = 6.61, *p* < .001] and explained 19% of the dependent variable. Daily RSVs were not related to daily new cases of COVID-19 [*F*(1, 253) = 0.00, *p* = .991, ${\eta}_p^2$ = .00, *β* = − .00], but negatively and moderately related to daily RSVs of new cases[*F*(1, 253) = 21.275, *p* < .001, ${\eta}_p^2$ = .08, *β* = − .43]. Daily RSVs for insomnia differed moderately among phases of managing the COVID-19 outbreak [*F*(5, 253) = 3.52, *p* = .004, ${\eta}_p^2$ = .07]. Daily RSVs for insomnia for each of the six phases of COVID-19 outbreak management are reported in Fig. 4. People were less likely to google insomnia before COVID-19 than during Phase 1. Finally, days of the week were not associated with differences in RSVs for insomnia [*F*(6, 253) = 1.50, *p* = .180, ${\eta}_p^2$ = .03].

## Discussion

To the best of our knowledge, this is the first study relying on search engine data to monitor the PD of the Italian population during the ongoing COVID-19 pandemic. First, we retrieved higher values for RSVs related to anxiety, than depression and insomnia. According to a study discussing the lifetime prevalence in the Italian population for psychological distress, the major depression and anxiety percentages were very similar (respectively 10.1 and 11.1%) [39]. Differences in information seeking for depression and anxiety terms may be related to the fact that depression is related to a loss of interest [40], so people affected by depression symptoms may be less prone to information-seeking behavior. As shown, we retrieved a significant effect of the pandemic phases on daily search queries. For insomnia, this effect is moderate, highlighting a difference between Pre-COVID and Phase 1: during the national lockdown, people searched the Internet more for information about insomnia, and this result confirmed other studies conducted during the pandemic [41], which demonstrated a worsening of insomnia symptoms and sleep quality.

The impact of pandemic phases on anxiety and depression searches is stronger, with unique and different patterns. Anxiety was searched more during the national lockdown, with a gradual decrease in the subsequent phases. This is also consistent with the results of a recent study consulting Google Trends to investigate variations in web searches for hygiene-preventive measures and emotional terms, such as “fear” and “stress”, from 1st January 2018 (i.e., 2 year before the outbreak of the pandemic) to the first 3 months of the COVID-19 outbreak in the Italian country [21]. While these findings highlighted positive phenomenon such as the increase of web interest in hygienic precautions (e.g., disinfectants and masks), they also called attention to the increase of the general interest in anxiety symptoms. Matching our results, also this study showed that web interest is always higher for anxiety than for depression. This is true if considering both the year before the pandemic and the early phases of the COVID-19 outbreak. Differently from this study, our work monitored RSVs for anxiety, depression, and insomnia until mid-October. Given that, we were able to detect that daily search for depression, instead, increased more slowly, with a peak in the period immediately after the national lockdown with the gradual loosening of the previous restrictions. We cautiously hypothesized that this information-seeking behavior is strictly related to the characteristics of anxiety and depression: facing a life-threatening danger, both real or also perceived, − as the ongoing pandemic and its related restrictions to working, social, and leisure activities – individuals can immediately and rapidly experience fear and anxiety as an evolutionary response to fight-or-flight. People focus on the dangerous and threatening situation, quickly recovering their energy and react by fighting the threat or escaping from it, with a response that could become pathological [42]. This evolutionary response also applies to the challenges of modern life. While the anxiety response is more immediate and rapid, the onset of depression symptoms could be slower [43], and anxiety symptoms could predict the onset of depressive symptoms [44]. In this particular context, maybe, after mobilizing the resources and failing in the response to the dangerous threat, people experience the exhaustion of their resources and a feeling of impotence, connected to depression. Phase 1 anxiety and fear, with the concurrent shock for the new and unknown situation, could be also considered as a risk factor for depression. This same could be for the peak of the search for “insomnia” during phase 1: insomnia symptoms could sometimes precede the onset of a depressive episode [45]. Moreover, some clinicians reported the “hut syndrome”: as the lockdown measures were lifted, people experienced difficulties in leaving their houses, where they felt protected and secure from the contagion; it was hard for them to return and re-adapt to their normal activities and they experienced depression as well. The presence of depressive symptomatology at the end of the lockdown is also highlighted in some studies, with higher levels of stress – such as the anxiety, fear, and shock experienced during phase 1 - and loneliness during lockdown leading to a detrimental effect on depression after lockdown [46].

We may think of searching for new cases also as a protective factor against depression and insomnia: staying informed about new cases could be seen as a sort of coping mechanism, focusing the attention on the threat and trying to get as much information about it, with a monitoring style [47] or dispositional vigilance [48]. Our results confirmed other studies conducted in the pandemic context and highlighting decreased mental health and the presence of depression, anxiety, sleep disturbances, Post Traumatic Stress Disorder symptoms, distress, fear, helplessness, worsened quality of life, both in the general population [23, 49–52] and in health care workers [53–55].

### Strengths and limitations

Certain limitations warrant mentioning. First, even though the number of people accessing the Internet is growing [56] and Google is the most widely-used search engine in Italy [10], our results are only related to Google users, and might not reflect the overall situation of the Italian population. Moreover, in considering our results and conclusions, it is important to bear in mind that these types of data “do not allow inference with the intention of the user (somebody looking for the keyword “cold” doesn’t necessarily have cold symptoms)” [8]. Thus, in our case, somebody searching for the term “depression” is not necessarily suffering from depression. This also adds to the fact that we did not have access to any users’ personal information related to the queries.

Another limitation is the fact that we considered only RSVs related to the Italian situation as a whole, not taking into account eventual regional variations (e.g., RSVs related to Lombardy, Sicily, Campania, Piedmont). This was done for at least two main reasons. First, we were interested in the Italian situation as a whole, not fragmented in the 20 regions across Italy, to better understand trends of PD in the first Western country severely hit by the pandemic. Second, the RSVs for regional areas were available as relative, not absolute data and, so, the comparison of PD trends across Italian regions is not possible. However, we are aware that considering the regional variations would have added finer shades of meaning and interpretation to the Italian “situations” related to PD and the first wave of the Covid pandemic.

Finally, there was a slight variation in the duration of the different phases of managing the COVID-19 outbreak, with the phase of the COVID-19 outbreak lasting only 16 days and other almost 2 months (e.g., Phase 1 and Second wave). Thus, because of this unequal phases length, our analysis might have undetected additional relevant differences in PD across them.

### Implications and future scope

The present study could have relevant implications for research and public health practices. First, applying this novel methodology, our study provides new and deep insights on population-level and longitudinal PD variation, while traditional studies usually focus on an individual level and with one or two-point measurement. Moreover, “people’s search terms reflect relatively uncensored desires for information and thus lack many of the biases of traditional self-report surveys” [20], which are usually implemented in this type of study focusing on mental health. From a methodological and research point of view, this study might contribute to the ongoing literature on infodemiology by contributing evidence regarding the feasibility of monitoring and investigating PD and its trends over time in the general population through information coming from Google Trends. Second, since in Italy we are, at the time of writing, approaching the third wave of the pandemic with new cases and new deaths surging, regional lockdown measures are being reintroduced, with millions of people facing another “phase 1”, and millions of other citizens dealing with other restrictions, as in the previous “phase 2” and “3”. We reasonably expect that the trends we retrieved during the first wave will re-occur, with a spike of information-seeking for anxiety and insomnia, followed by depression. Maybe, mental health in this new wave will be worsened by the presence of exhausted personal resources, or, conversely, will be benefited by the previous development of ad hoc coping strategies.

We believe that our data could be useful to inform health policies and institutions, both a posteriori and in real-time. This RSVs analysis could be indeed implemented as a sort of active surveillance, to provide dynamic and simultaneous information about the symptoms most searched at the moment and the need of the populations. Future studies are warranted, approaching this active surveillance. Moreover, more studies should be implemented, analyzing RSVs jointly with other users’ personal information – such as geographical location, the intention of the user, real presence (or absence) of symptoms and psychological distress searched. This could help in providing timely and tailored interventions, both at the individual and population/public health level, to improve psychological wellbeing and to mitigate the costly effects on mental health and the other aspects of the pandemic [3]. This pertains not only to this posited third wave but also to similar situations in the future. Psychological support, psychoeducation, prevention, and coping strategies, and psychotherapy sessions could be implemented, in which the advantages of the new e-health technologies are drawn upon [57–59]. Such technologies have proven to be useful in the healthcare context [60], overcoming and respecting the restraints imposed by the necessary social distancing measures.

## Conclusions

Our study used a novel methodology to gain insights on the population and longitudinal level variation of mental PD in the Italian population across the different phases of the COVID-19 pandemic. We retrieved differences in Google Trend queries of mental health-related words during the ongoing pandemic, with a peak of searches for “insomnia” and “anxiety” in the lockdown phases and “depression” in the immediately subsequent phase with a gradual loosening of the previous restrictions. Our results provide a precise picture of the online information-seeking behaviour regarding mental health during this unique situation; they could be useful to inform health policies and institutions, thereby promoting timely and tailored intervention to improve psychological wellbeing in the population.

## Supplementary Information

**Additional file 1.** Frequency of search terms considered from Google Trends website.

## Data Availability

The datasets used and/or analysed during the current study are available from the corresponding author on reasonable request.

## Abbreviations

COVID-19: Coronavirus disease 2019
PD: Psychological distress
RSV: Relative search volume
