# Supplementary material for: When in doubt, Google it: distress-related information seeking in Italy during the COVID-19 pandemic
Source: BMC Public Health. 2021 Oct 20;21:1902. doi: 10.1186/s12889-021-11887-2 (PMC8528555; doi:10.1186/s12889-021-11887-2)
Supplement: Supplementary file 1 — Additional file 1. Frequency of search terms considered from Google Trends website. [file 12889_2021_11887_MOESM1_ESM.pdf]

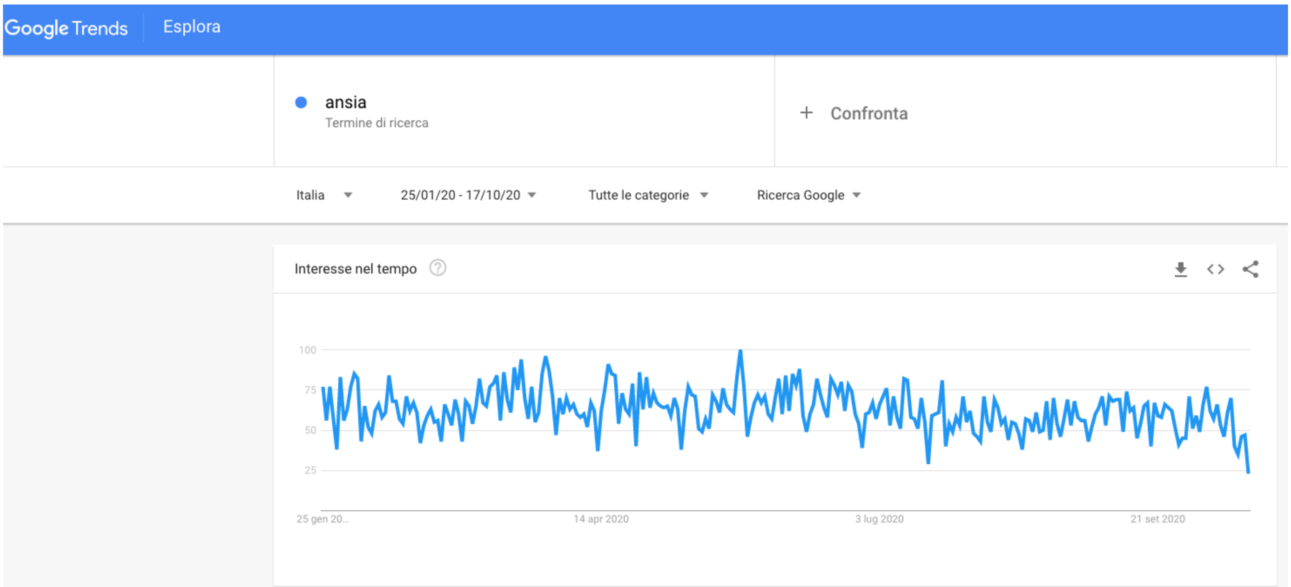
Google Trends
Esplora
ansia
Termine di ricerca
Confronta
Italia
25/01/20 - 17/10/20
Tutte le categorie
Ricerca Google
Interesse nel tempo
100
75
50
25
25 gen 20...
14 apr 2020
3 lug 2020
21 set 2020

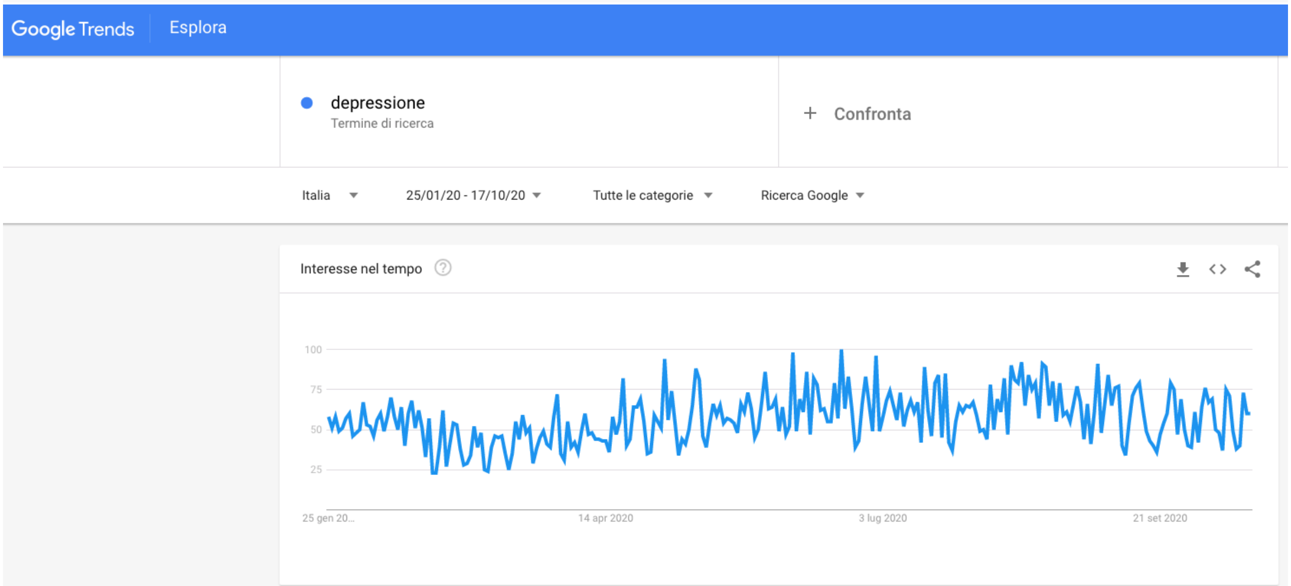
Google Trends
Esplora
depressione
Termine di ricerca
Confronta
Italia
25/01/20 - 17/10/20
Tutte le categorie
Ricerca Google
Interesse nel tempo
100
75
50
25
25 gen 20...
14 apr 2020
3 lug 2020
21 set 2020

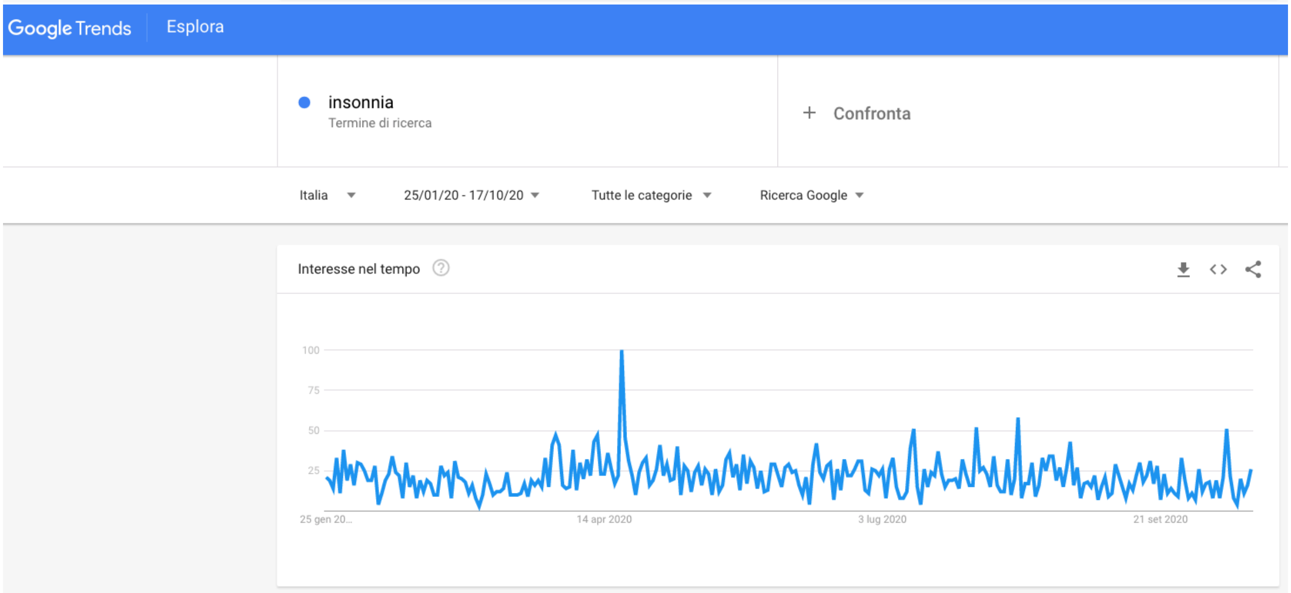
Google Trends
Esplora
insonnia
Termine di ricerca
Confronta
Italia
25/01/20 - 17/10/20
Tutte le categorie
Ricerca Google
Interesse nel tempo
100
75
50
25
25 gen 20...
14 apr 2020
3 lug 2020
21 set 2020

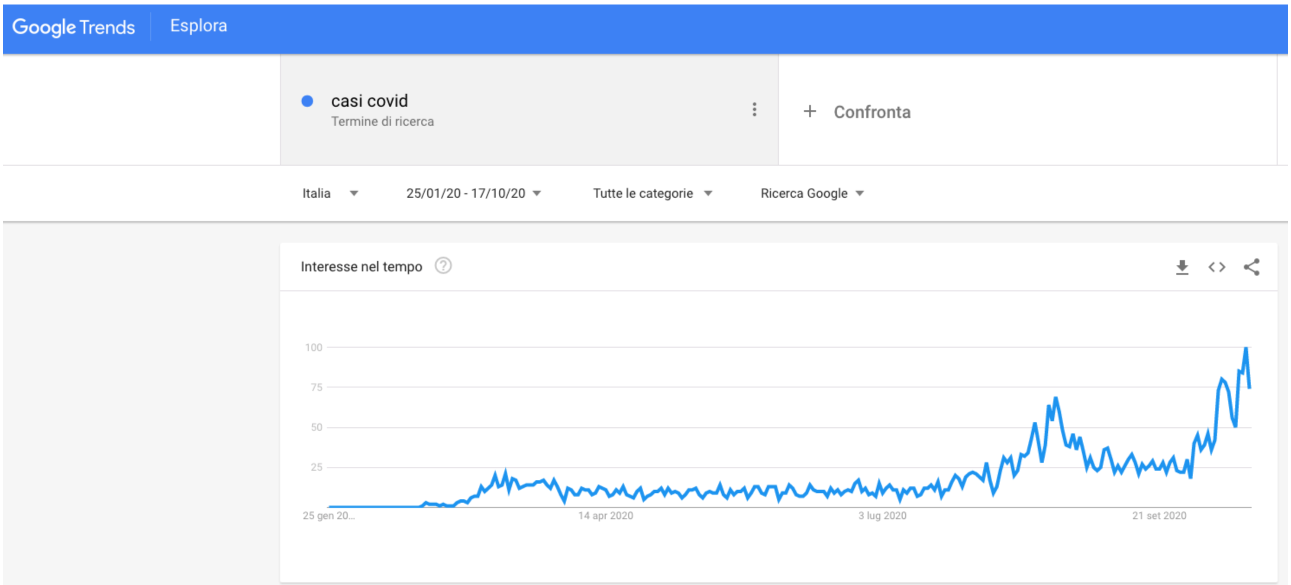
Google Trends
Esplora
casi covid
Termine di ricerca
Confronta
Italia
25/01/20 - 17/10/20
Tutte le categorie
Ricerca Google
Interesse nel tempo
100
75
50
25
25 gen 20...
14 apr 2020
3 lug 2020
21 set 2020

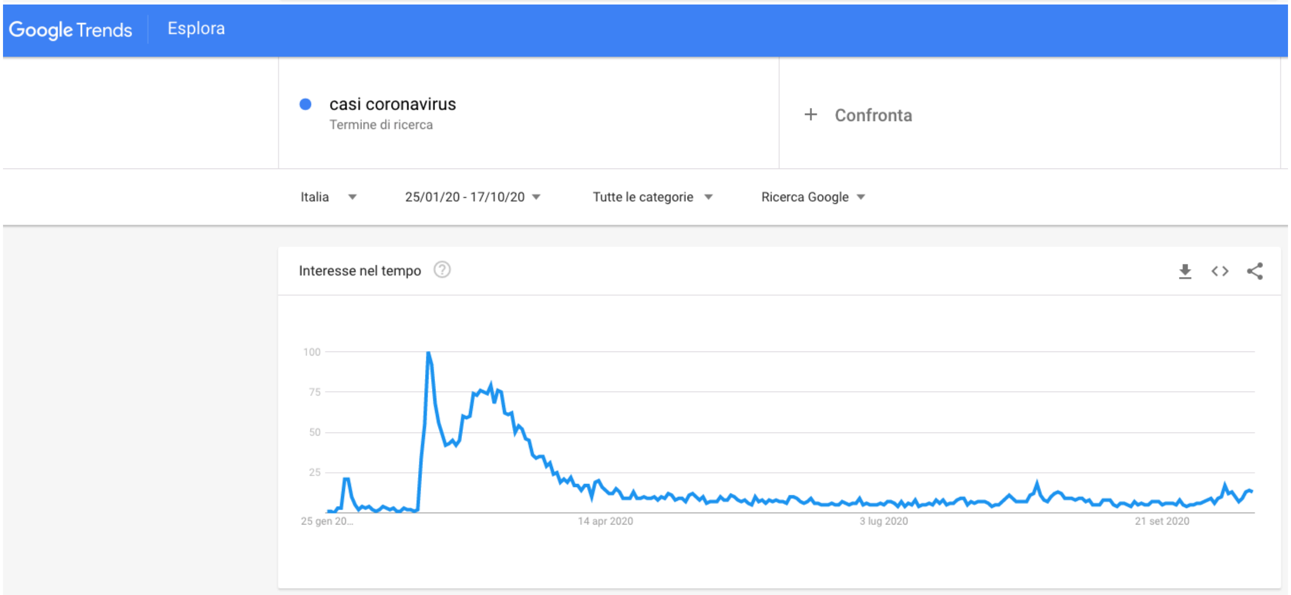
Google Trends
Esplora
casi coronavirus
Termine di ricerca
Confronta
Italia
25/01/20 - 17/10/20
Tutte le categorie
Ricerca Google
Interesse nel tempo
100
75
50
25
25 gen 20...
14 apr 2020
3 lug 2020
21 set 2020
